# The longitudinal association between artificial sweetener intake and the risk of type 2 diabetes among adults aged 18 years and older: a systematic review and meta-analysis

**DOI:** 10.3389/fnut.2026.1886458

**Published:** 2026-08-11

**Authors:** Siyuan Lu, Yi Lu, Rui Xu, Wenfeng Hou, Ting Xu, Wei Tang

**Affiliations:** 1 School of Management, Xuzhou Medical University, Xuzhou, China; 2 First Clinical Medical College, Xuzhou Medical University, Xuzhou, China; 3 School of Public Health, Xuzhou Medical University, Xuzhou, China; 4 Dept. of Endocrinology, Geriatric Hospital of Nanjing Medical University, Nanjing, China

**Keywords:** artificial sweetener, cohort, meta-analysis, prospective study, type 2 diabetes mellitus

## Abstract

**Background:**

Artificial sweeteners are commonly used as sugar substitutes because they provide sweetness with little or no caloric content. However, the relationship between artificial sweetener consumption and type 2 diabetes (T2D) remains controversial. This meta-analysis was conducted to investigate the longitudinal association between artificial sweetener intake and T2D among adults.

**Methods:**

Electronic databases in English and Chinese were searched for prospective studies published from inception to March 2025. Using a random-effects model, the pooled hazard ratios (HRs) and 95% confidence intervals (CIs) were calculated to investigate the longitudinal association between artificial sweetener intake and T2D among adults aged 18 years and older. Between-study heterogeneity was assessed using the *I*^2^ statistic, while the robustness of the findings was examined using a leave-one-out sensitivity analysis. Publication bias was evaluated by inspection of funnel plots and Egger’s regression test.

**Results:**

A total of six cohort studies including 721,003 participants were analyzed in this meta-analysis. Compared with those with low or no consumption of artificial sweeteners, participants with higher artificial sweetener intake were more likely to develop T2D (pooled HR = 1.31, 95% CI = 1.16–1.49). Substantial heterogeneity was observed across studies (*I*^2^ = 86.02%), while the overall estimate remained stable in sensitivity analysis. No significant publication bias was observed among the studies included in this meta-analysis.

**Conclusion:**

A longitudinal association was observed between artificial sweetener intake and the risk of developing T2D among adults. This study has important public health implications. The findings of this study highlight that artificial sweeteners may not be a completely risk-free alternative to sugar in dietary interventions aimed at reducing high-energy sugar intake and suggest that their potential adverse metabolic effects should be considered in public health policy-making and clinical dietary recommendations regarding artificial sweetener intake.

## Introduction

Over the past few decades, the global health landscape has been profoundly affected by the rising prevalence of type 2 diabetes (T2D) (1). It has been documented that more than 500 million adults are currently living with diabetes worldwide, and the number is projected to increase to approximately 700 million by 2045, of whom approximately 90% will have T2D (1). Additionally, the increasing prevalence of T2D imposes substantial economic and healthcare burdens worldwide (2). In China, the age-standardized prevalence of T2D among adults was estimated to be approximately 13.7% in 2023 based on nationally representative data, with a considerable proportion of cases remaining undiagnosed (3).

With growing concerns about the unfavorable health consequences of excessive sugar intake, artificial sweeteners have been widely used as low- or non-caloric alternatives in beverages, dairy products, and processed foods (4). Artificial sweeteners typically include aspartame, sucralose, saccharin, and acesulfame potassium. They are frequently consumed by individuals with excess body weight or abnormal metabolic conditions as a strategy to reduce overall energy intake and improve glycemic control (5). However, the long-term metabolic effects of artificial sweetener consumption remain unclear, as inconsistent findings have been documented in previous studies (6–15). Moreover, the guidelines released by the World Health Organization (WHO) in 2023 stated that artificial sweeteners should not be used as a long-term strategy for weight control or the prevention of non-communicable diseases (NCDs) (16). The WHO guidelines have also indicated that artificial sweetener intake may have limited benefits for sustainable weight management, and that long-term use may lead to adverse health outcomes (16).

Although the association between artificial sweetener intake and metabolic disorders such as T2D remains controversial, several potential mechanisms have been proposed to explain the possible association between them (17, 18). First, artificial sweeteners may alter both the composition and metabolic activity of intestinal microbial communities, subsequently affecting host glucose metabolism and insulin sensitivity (7, 19). Such alterations in the gut microbiota may further influence microbial metabolites, intestinal barrier function, and low-grade inflammatory responses, ultimately contributing to impaired glucose tolerance and broader metabolic dysregulation (7, 19, 20). Second, long-term exposure to intense sweetness in the absence of energy intake may disrupt the learned physiological responses to sweet taste and subsequently weaken the normal predicative association between sweetness and caloric content (17, 21). This may further influence appetite regulation, food preference, compensatory eating behavior, and glucose homeostasis, thereby affecting long-term energy balance and metabolic health. Finally, artificial sweeteners may also affect metabolic regulation through neural and hormonal pathways involved in appetite control and glucose processing, as the activation of sweet taste receptors in the oral cavity and gastrointestinal tract may influence endocrine signaling related to insulin secretion, incretin responses, and satiety regulation (17, 21).

Considering the potential mechanisms linking artificial sweetener intake and T2D, the previously inconclusive evidence regarding their association, and the concerns raised by WHO (6–16), there is a need to further examine the long-term association between artificial sweetener consumption and T2D in large-scale adult populations using longitudinal data. Therefore, to investigate the potential longitudinal relationship between long-term intake of artificial sweeteners and T2D, we conducted this systematic review and meta-analysis of prospective follow-up studies.

## Methods

### Study design and literature search strategy

For this systematic review and meta-analysis, articles published in English or Chinese were retrieved through electronic databases, including PubMed, Embase, Web of Science, and China National Knowledge Infrastructure (CNKI). The Medical Subject Headings (MeSH) terms and free-text terms of “artificial sweetener,” “non-nutritive sweetener,” “low-calorie sweetener,” “type 2 diabetes mellitus,” “T2D,” and “diabetes” were used as keywords for searching articles published from inception to 31 March 2025. Moreover, the following Boolean operators were employed for the literature search: (“artificial sweetener” OR “non-nutritive sweetener” OR “low-calorie sweetener”) AND (“type 2 diabetes” OR T2D OR diabetes).

**Figure 1:**
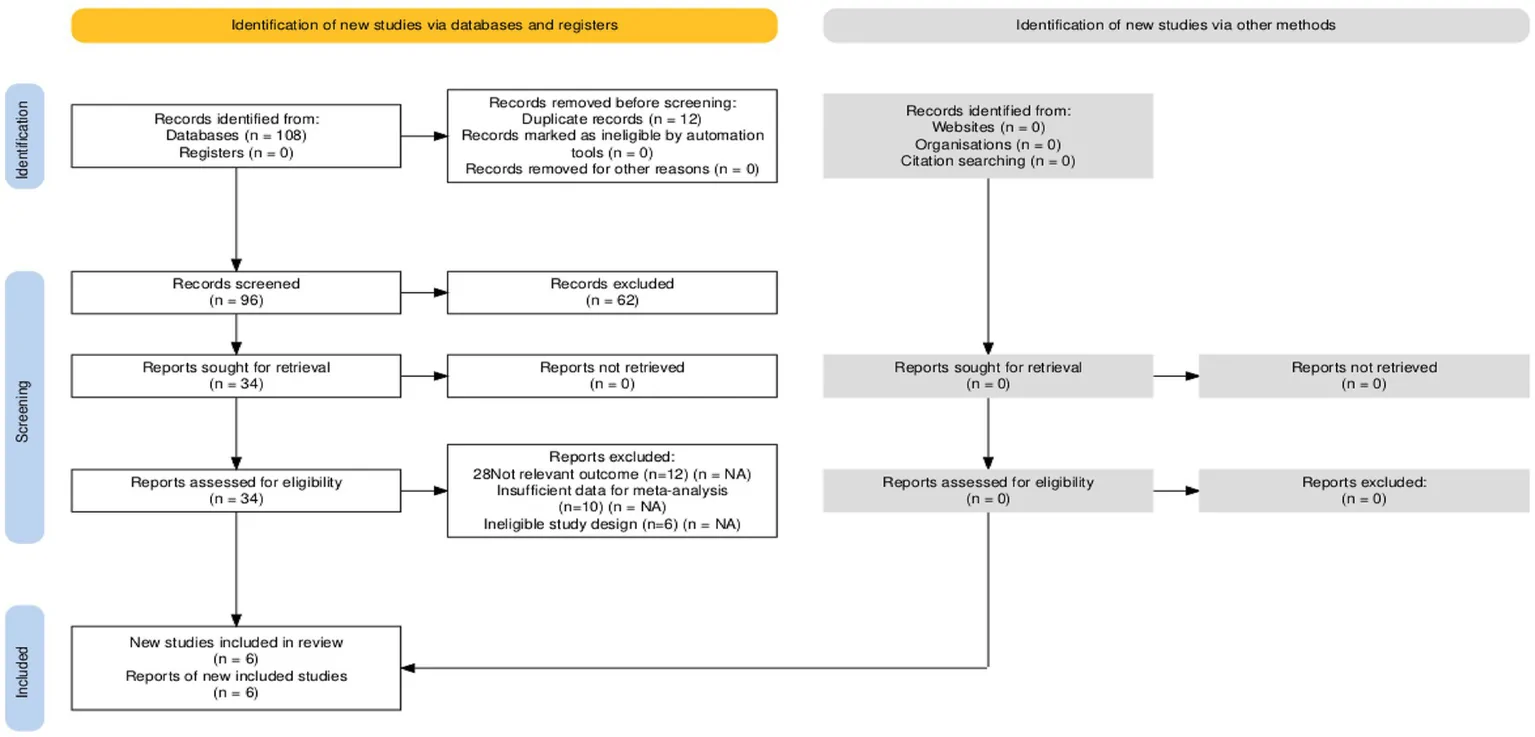
PRISMA 2020 flow diagram of the study selection.

The study was performed in accordance with the guidelines recommended in the Preferred Reporting Items for Systematic Reviews and Meta-Analyses (PRISMA) statement (22). In addition, the reference lists of all included studies were manually screened to identify potentially relevant articles (23). The flow chart of the search strategy is shown in Figure 1. Only publicly available second-hand data without identification were retrieved and analyzed; thus, ethical approval was waived.

### Inclusion and exclusion criteria

Studies were included in this systematic review and meta-analysis if they met the following criteria: (1) prospective observational studies, (2) original population-based studies, (3) participants aged 18 years and older, (4) artificial sweeteners (including overall intake or specific types) that were defined clearly, (5) T2D, as the outcome variable, was clearly determined based on the WHO 1999 or ADA diagnostic criteria (16, 24), and (6) the association between artificial sweetener intake and T2D was examined, with effect estimates presented. Additionally, no minimum sample size threshold was pre-specified for study inclusion.

Studies that did not meet the above-mentioned inclusion criteria were excluded from this meta-analysis. Moreover, the following articles were also excluded: reviews, commentaries, conference abstracts, and studies with insufficient information for statistical analysis. For publications based on the same study population, only the study with the largest sample size, longest follow-up duration, or the most complete information was included in the meta-analysis (24).

### Data extraction

All relevant articles were reviewed independently by two researchers. Each article was screened first by title and abstract, and then the full text was retrieved for further assessment. Publications were included in this study only when the two independent researchers reached unanimous agreement. If disagreements occurred, another independent researcher was invited to participate in the discussion. The final inclusion decision was based on the consensus of at least two of the three independent researchers.

For the included articles, data were also extracted independently by two researchers using the same extraction approaches. The extracted information included the following: first author, year of publication, study location, participant number, sampling approach, participants’ age and sex, follow-up period and follow-up rate, independent and outcome variables’ assessment, statistical estimates, and 95% confidence intervals (CIs).

### Quality assessment

The quality of the included studies was evaluated using the Newcastle–Ottawa Scale (NOS). This scale assesses study quality based on three domains: selection of study participants, comparability of study groups, and assessment of outcomes. The total score ranges from 0 to 9, with a score of ≥7 indicating high quality and a score of <7 indicating low quality (25).

### Statistical analysis

All six finally included cohorts reported the effect size as hazard ratios (HRs) (9–14). Therefore, HRs and 95% CIs were used as the effect measures in this meta-analysis (26). The DerSimonian–Laird random-effects model was employed to calculate pooled effect estimates, which were presented with forest plots (27). The heterogeneity among studies was assessed using Cochran’s *Q* test and the *I*^2^ statistic. An *I*^2^ value of > 50% or a *Q*-test *p*-value of < 0.10 was considered significantly heterogeneous. Moreover, between-study variance (*τ*^2^) was also computed to assess the heterogeneity. Next, sensitivity analysis was conducted by sequentially excluding each included study using a leave-one-out approach to evaluate the robustness of the pooled effect estimates. Moreover, funnel plots and Egger’s regression test were employed to investigate publication bias (28). Additionally, the Grading of Recommendations Assessment, Development and Evaluation (GRADE) framework was used to assess the certainty of the evidence examined in this meta-analysis. A two-sided *p*-value of <0.05 was set as the significance level. All statistical analyses were performed using Stata version 17 (StataCorp, College Station, TX, USA).

## Results

### Characteristics of included studies

A total of 96 relevant original articles were initially retrieved and screened by title and abstract. Then, 34 articles were assessed for eligibility through full-text review. Of these, six prospective studies published between 2009 and 2025 were finally included in the quantitative synthesis. For the pooled data, the average follow-up period was 10.1 years, with a sample size of 721,003. Among the six included studies, the shortest follow-up period was 5 years and the longest was 20 years, while the smallest sample size was 6,814 and the largest was 340,234. These analyzed longitudinal studies were conducted in the USA, Europe, and Japan. Additionally, a total of 38,445 incident T2D cases were identified during 7,280,376 person-years of follow-up, yielding a crude incidence density of 5.28 cases per 1,000 person-years in this pooled longitudinal dataset.

Table 1 displays the detailed study characteristics and effect estimates. Of the six included prospective studies, Debras et al. analyzed data from the French NutriNet-Santé cohort and reported a significantly positive association between artificial sweetener consumption and T2D risk (9). In the European EPIC-InterAct study, Romaguera et al. examined the association between artificial sweetener beverage consumption and incident T2D (10). Nettleton et al. used data from the Multi-Ethnic Study of Atherosclerosis (MESA) in the United States to investigate the association between artificially sweetened soda consumption and the risks of metabolic syndrome and T2D (11). Sakurai et al. evaluated the associations between sugar-sweetened and artificially sweetened beverage consumption and diabetes risk using the Japanese Public Health Center-based (JPHC) cohort (12). de Koning et al. analyzed data from the Health Professionals Follow-up Study (HPFS) to assess long-term associations between artificially sweetened beverage consumption and metabolic disease risk (13). More recently, Pacheco et al. further examined the association between artificially sweetened beverage intake and incident T2D using data from three large cohorts in the USA—the Nurses’ Health Study (NHS), Nurses’ Health Study II (NHS II), and the Health Professionals Follow-up Study (HPFS) (14).

**Table 1:** Characteristics of the included studies and main outcome estimates.

First author (year)	Country/cohort	Exposure	Comparison	Outcome	Sample size (*N*)	Cases (*n*)	Follow-up (years)	Effect (HR, 95% CI)	BMI adjusted	Main covariates	Nos
Debras et al. (2023) (9)	France (NutriNet-Santé)	Total artificial sweetener	Highest *vs*. lowest	Incident T2DM	105,588	972	9.1	1.69 (1.45–1.97)	Yes	Age, sex, BMI	8
Romaguera et al. (2013) (10)	Europe (EPIC-InterAct)	Artificially sweetened beverages	≥1 serving/day *vs*. <1/month	Incident T2DM	340,234	12,403	11.3	1.18 (1.07–1.30)	Yes	Age, BMI, lifestyle	8
Nettleton et al. (2009) (11)	USA (MESA)	Artificially sweetened soda	Daily *vs*. none	Incident T2DM	6,814	413	7.0	1.36 (1.11–1.66)	Yes	Age, BMI, energy intake	7
Sakurai et al. (2014) (12)	Japan (JPHC)	Artificially sweetened beverage and sugar-sweetened and	High *vs*. low	Incident T2DM	36,173	2,037	5.0	1.43 (1.12–1.82)	Yes	Age, BMI, lifestyle	8
de Koning et al. (2011) (13)	USA (HPFS)	Artificially sweetened beverages	High *vs*. low	Incident T2DM	40,389	2,680	20.0	1.31 (1.15–1.49)	Yes	Age, BMI, diet	8
Pacheco et al. (2025) (14)	USA (NHS, NHSII, HPFS)	Artificially sweetened beverages	≥2 servings/day *vs*. <1/month	Incident T2DM	191,805	19,940	7.5	1.11 (1.07–1.16)	Yes	Age, smoking, alcohol, and physical activity	8

Definitions of “high *vs*. low” intake were based on the criteria reported in the original studies (see Methods section for details).

HR, hazard ratio. The effect estimates were presented based on the fully-adjusted models reported in the original studies.

With regard to exposure measurement, validated dietary assessment instruments, specifically food frequency questionnaires (FFQs), were used to estimate the intake level of artificial sweeteners in all of the studies included in this meta-analysis. For outcome assessment, all studies defined incident T2D as the primary endpoint. Diabetic cases were identified through self-report by participants and then confirmed with medical records, clinically standardized diagnostic criteria, or national disease registries. Each study recorded multivariable-adjusted effect estimates, together with corresponding 95% CIs, by comparing the highest level of artificial sweetener intake with the lowest or non-consumption group. According to the Newcastle–Ottawa Scale (NOS), the methodological quality of the included studies ranged from 7 to 8 points, indicating overall high quality.

### The association between artificial sweetener intake and the risk of T2D

**Figure 2:**
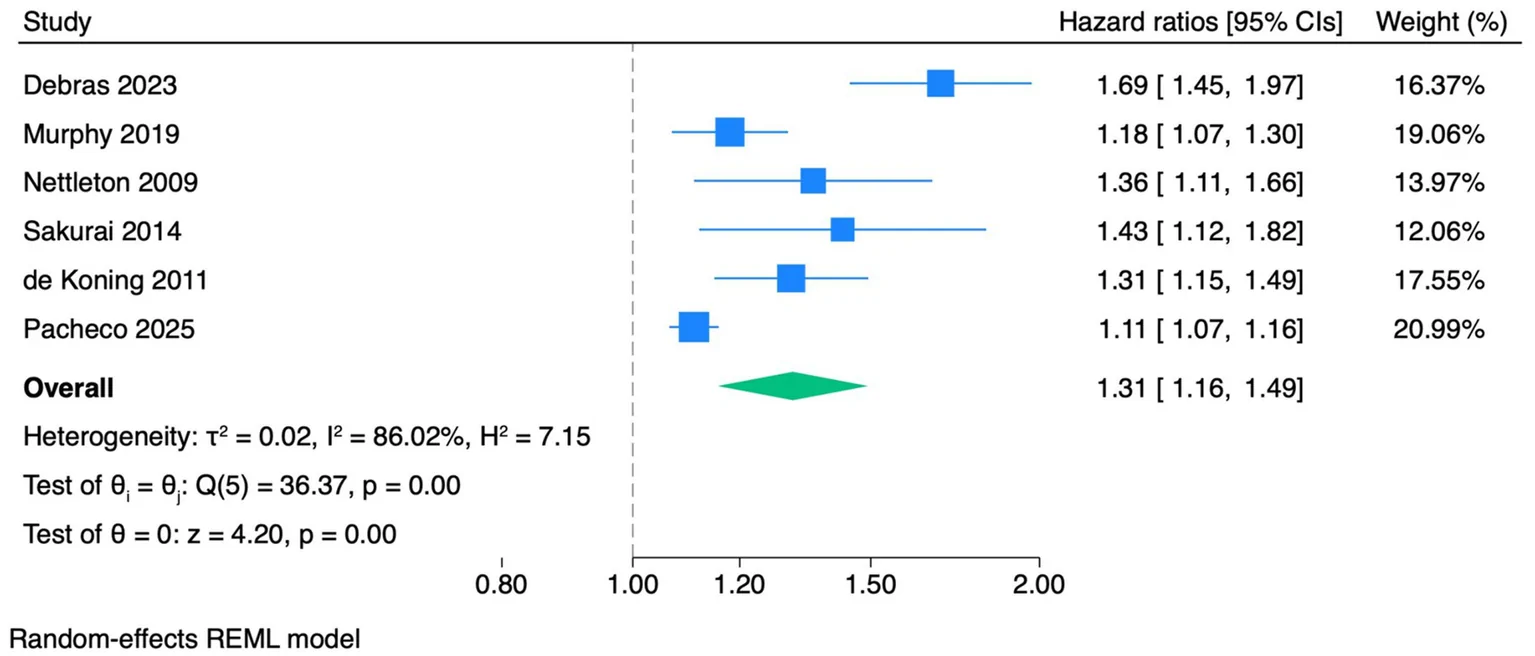
The longitudinal association between artificial sweetener intake and the risk of developing type 2 diabetes among adults aged 18 years and older.

Figure 2 presents the pooled effect estimates regarding the longitudinal association between artificial sweetener intake and T2D among adults. Overall, substantial heterogeneity was observed among the included studies (Cochran’s *Q* = 36.37, df = 5, *p* < 0.001; *I*^2^ = 86.02%). Moreover, the between-study variance was *τ*^2^ = 0.02, with the 95% prediction interval ranging from 0.97 to 1.78. Thus, a random-effects model, which is an appropriate analysis approach, was employed to calculate the conservative pooled estimate.

Based on the pooled data, long-term artificial sweetener intake was significantly positively associated with the risk of developing T2D (HR = 1.31, 95% CI = 1.16, 1.49) among adults aged 18 years and older. Importantly, all six longitudinal studies consistently reported positive associations between artificial sweetener intake and T2D (9–14), with the largest effect size (HR = 1.69, 95% CI = 1.45, 1.97) observed in the study by Debras et al. (9) and the smallest (HR = 1.11, 95%CI = 1.07, 1.16) in the study by Pacheco et al. (14).

### Sensitivity analysis

To examine the robustness of the pooled effect, each included study was sequentially excluded, and the pooled effect estimate was recalculated in this analysis. The results showed that the direction of the association remained unchanged after the omission of any single study, and the pooled effect estimates ranged from 1.23 (95%CI = 1.12, 1.34) to 1.37 (95%CI = 1.21, 1.55), indicating that the overall findings were stable and robust in this meta-analysis.

### Publication Bias

**Figure 3:**
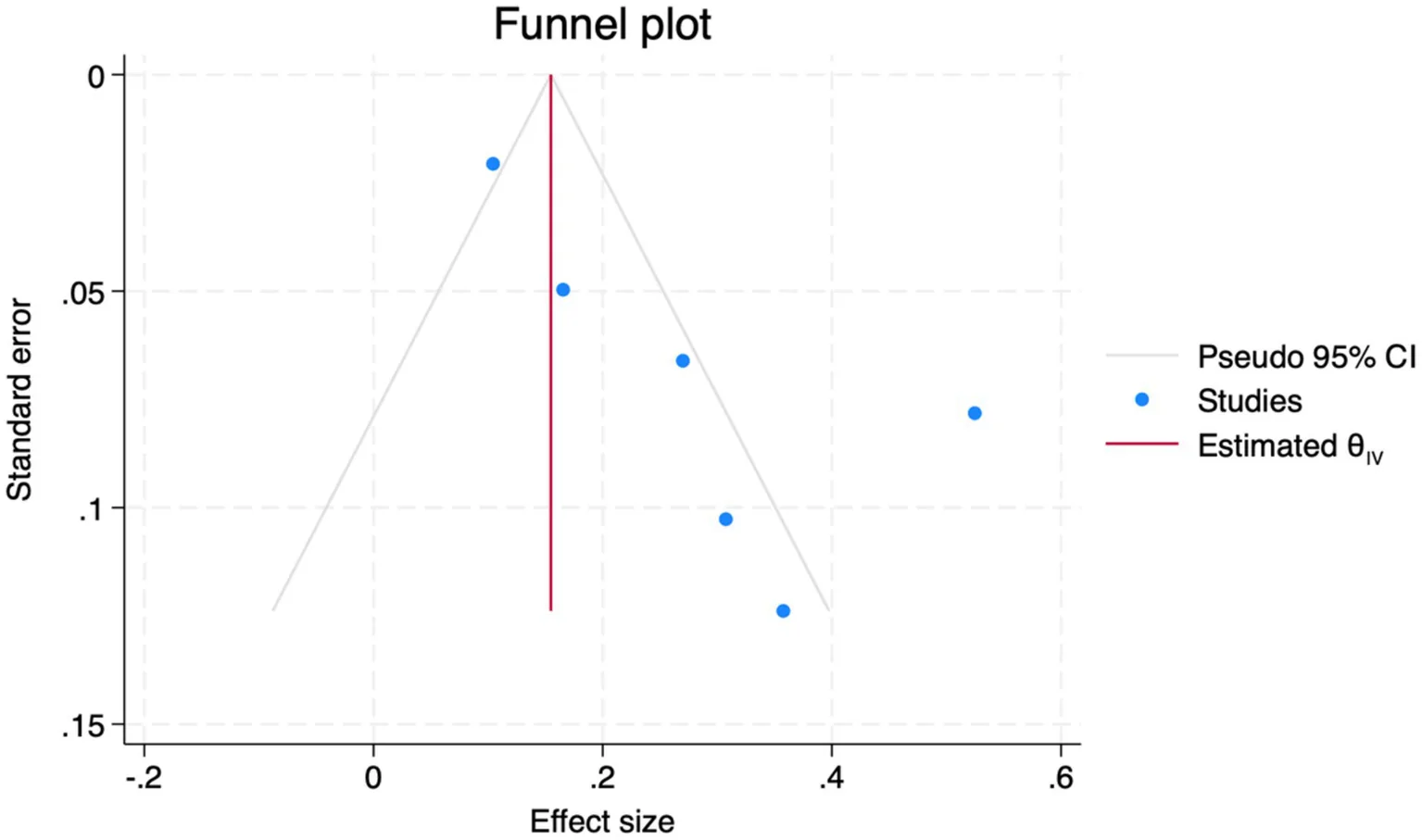
Funnel plot assessing publication bias among the included studies.

Figure 3 demonstrates the publication bias using a funnel plot. The distribution of the included studies appeared symmetrical around the pooled effect estimate. Additionally, Egger’s regression test was also performed to evaluate potential publication bias, showing no significant evidence of small-study effects (*p* > 0.05). Thus, there was no obvious publication bias for this meta-analysis.

### Certainty of evidence

Using the GRADE assessment approach, the certainty of evidence was rated as low in this meta-analysis, although a significantly positive association was observed between artificial sweetener intake and incident T2D. The details are presented in Table 2.

**Table 2:** GRADE summary of findings for the association between artificial sweetener intake and incident type 2 diabetes in this meta-analysis study.

Outcome	Participants (studies)	Relative effect	Certainty of evidence	Comments
Incident type 2 diabetes	721,003 participants (6 prospective cohort studies)	HR 1.31 (95% CI: 1.16–1.49)	Low	The low certainty of evidence may be attributed to limited number of studies included, observational study design, and/or substantial between-study heterogeneity

## Discussion

This meta-analysis, based on pooled prospective observational studies, found a significant positive association between long-term artificial sweetener consumption and the risk of developing T2D among adults aged 18 years and older. The sensitivity analysis indicated that such a positive association remained stable across the included studies. The findings suggest that artificial sweeteners may not be a metabolically neutral alternative to sugar in strategies aimed at reducing dietary energy intake.

Previous studies have reported inconsistent findings on the relationship between artificial sweetener intake and metabolism-related conditions (15, 20, 29–31). Some studies found no association between artificial sweetener consumption and T2D (30, 31), whereas other studies have reported positive associations between them, similar to the association observed in the present study (20, 21, 29). These discrepancies may be attributable to differences in study design, exposure assessment, and the covariates included in the analyses. Notably, the studies that reported no association between artificial sweetener intake and T2D were randomized controlled trials, which usually focused on short-term artificial sweetener intake and metabolic outcomes, and thus did not detect significant adverse metabolic effects of long-term artificial sweetener intake (30, 31). By contrast, a positive relationship between artificial sweetener consumption and T2D was observed in long-term prospective observational studies, which captured consumption patterns of artificial sweeteners and their adverse health outcomes over a long time period (20, 21, 29). Therefore, considering the nature of the study design, differences in the association between artificial sweetener intake and metabolism-related conditions observed between short-term randomized controlled trials and long-term prospective observational studies should be interpreted with caution and objectively rather than simply regarded as direct conflicting evidence.

There are several potential mechanisms underlying the positive long-term association between artificial sweetener intake and T2D. First, artificial sweeteners may alter the composition and functional activity of the gut microbiota, thereby affecting host glucose tolerance and insulin sensitivity (20). Second, artificial sweeteners may stimulate intestinal sweet taste receptors and thereby influence the secretion of incretin hormones, such as glucagon-like peptide-1 (GLP-1), potentially disrupting normal glucose regulation (29). Additionally, long-term exposure to sweet taste without corresponding caloric delivery may modify sweet taste perception, reward pathways, and habitual dietary behaviors, thereby influencing appetite regulation and energy intake patterns (21). Collectively, artificial sweetener intake-related behavioral and physiological pathways may, at least partly, contribute to metabolic dysregulation.

The heterogeneity (*I*^2^ = 86.02%) was substantial across the studies included in this meta-analysis. Therefore, a random-effects model, which is appropriate, was employed to calculate the pooled effect estimates. Moreover, further analyses were conducted to explore potential contributors to heterogeneity. It was observed that exposure definition made a significant contribution to heterogeneity. The heterogeneity was significantly reduced from 86.25 to 68.8% (*p* < 0.001) when exposure variables were restricted to artificially sweetened beverages or diet soda. On the other hand, the contribution to the heterogeneity by study population or follow-up duration alone was not statistically significant, although each factor appeared to reduce between-study heterogeneity. Therefore, the substantial between-study heterogeneity was influenced primarily by exposure definition, together with other potential factors. This highlights that the findings should be interpreted cautiously.

This study has significant public health implications. Currently, artificial sweeteners have been widely used as an alternative strategy to reduce high-energy sugar intake for weight control and diabetes prevention, although the metabolic safety of long-term artificial sweetener intake remains an important health concern (4, 5). The findings of this study suggest that participants who consume artificial sweeteners over a long period may be at an elevated risk of developing T2D. This implies that artificial sweetener intake may not be a risk-free option for individuals seeking to prevent weight gain or manage blood glucose levels. The potentially adverse metabolic effects should therefore be carefully considered when formulating dietary recommendations regarding artificial sweetener consumption. In the future, further research is encouraged to investigate the longitudinal associations between specific types of, in addition to the overall, artificial sweetener intake and T2D. Moreover, long-term target intervention emulation studies may be of help for further elucidating the “true” relationship between artificial sweetener intake and T2D (32).

There are several strengths to this study. First, only prospective studies were included in this meta-analysis, allowing examination of the long-term longitudinal association between artificial sweetener consumption and T2D. Second, the pooled sample size was more than 700,000, and the average follow-up duration was longer than 10 years, providing sufficient statistical power and stability. Third, the outcome event was determined clearly and consistently across the included cohorts. Finally, validated instruments were used to estimate artificial sweetener consumption.

Several limitations are worth mentioning. First, artificial sweetener is usually used for body weight control or/and improvement of metabolic health (4, 5). Individuals already at an elevated metabolic risk may be more likely to consume artificial sweetener as a dietary modification, which may raise the possibility of reverse causation between artificial sweetener intake and metabolism-related conditions (e.g., obesity, prediabetes, or metabolic syndrome) (33, 34). In fact, users of artificial sweeteners may be those already at high risk of T2D at baseline. However, no direct reverse causation exists for the association between artificial sweetener intake and T2D in this meta-analysis because all included studies were longitudinal investigations, with all T2D patients at baseline excluded and only newly incident T2D cases considered as the outcome event. Second, although classical confounders, such as body weight, were adjusted for in the studies included in this meta-analysis, residual confounding cannot be fully excluded (32). Third, only six longitudinal studies were included in this meta-analysis, and they had substantial heterogeneity. These limitations imply that the pooled estimates may be potentially unstable. Therefore, the findings shall be interpreted cautiously. Fourth, low certainty of evidence was rated for the findings examined in this meta-analysis, which may be due to the small number and observational nature of the studies analyzed and substantial between-study heterogeneity. This also highlights the prudence when interpreting the positive association between artificial sweetener intake and T2D examined in the present study. Fifth, this meta-analysis was not registered, and thus, a PROSPERO registration number was not available. Finally, exposure information was self-reported, implying potential recall bias, although validated instruments were employed. Therefore, the findings in this study should still be interpreted with caution, even though they were examined based on long-term prospective data.

## Conclusion

A positive association between artificial sweetener consumption and the risk of developing T2D among adults aged 18 years and older was observed based on long-term longitudinal data. This study has important public health implications. The findings highlight that artificial sweeteners may not be a completely risk-free alternative to sugar in dietary interventions aimed at reducing high-energy sugar intake and suggest that their potential adverse metabolic effects should be considered in policy-making and clinical dietary recommendations regarding artificial sweetener intake.

## Data Availability

The original contributions presented in the study are included in the article/supplementary material; further inquiries can be directed to the corresponding author.
